# WGS-Based Analysis of Carbapenem-Resistant *Acinetobacter baumannii* in Vietnam and Molecular Characterization of Antimicrobial Determinants and MLST in Southeast Asia

**DOI:** 10.3390/antibiotics10050563

**Published:** 2021-05-11

**Authors:** Gamal Wareth, Jörg Linde, Ngoc H. Nguyen, Tuan N. M. Nguyen, Lisa D. Sprague, Mathias W. Pletz, Heinrich Neubauer

**Affiliations:** 1 Friedrich-Loeffler-Institute, Institute of Bacterial Infections and Zoonoses (IBIZ), Naumburger str.96a, D-07743 Jena, Germany; Joerg.linde@fli.de (J.L.); lisa.sprague@fli.de (L.D.S.); heinrich.neubauer@fli.de (H.N.); 2 Institute for Infectious Diseases and Infection Control, Jena University Hospital, Am Klinikum 1, D-07747 Jena, Germany; Mathias.Pletz@med.uni-jena.de; 3 Faculty of Veterinary Medicine, Benha University, Moshtohor, Toukh 13736, Egypt; 4 Phutho Department of Healthcare, Phutho 35124, Vietnam; 5 Phutho General Hospital, Phutho 35125, Vietnam; minhtuannn@hvu.edu.vn

**Keywords:** *Acinetobacter baumannii*, carbapenem-resistant, MDR, WGS, Vietnam, Southeast Asia

## Abstract

Carbapenem-resistant *Acinetobacter baumannii* (*A. baumannii*, CRAb) is an emerging global threat for healthcare systems, particularly in Southeast Asia. Next-generation sequencing (NGS) technology was employed to map genes associated with antimicrobial resistance (AMR) and to identify multilocus sequence types (MLST). Eleven strains isolated from humans in Vietnam were sequenced, and their AMR genes and MLST were compared to published genomes of strains originating from Southeast Asia, i.e., Thailand (*n* = 49), Myanmar (*n* = 38), Malaysia (*n* = 11), Singapore (*n* = 4) and Taiwan (*n* = 1). Ten out of eleven Vietnamese strains were CRAb and were susceptible only to colistin. All strains harbored *ant*(3”)-IIa, *arm*A, *aph*(6)-Id and *aph*(3”) genes conferring resistance to aminoglycosides, and *bla*OXA-51 variants and *bla*ADC-25 conferring resistance to ß-lactams. More than half of the strains harbored genes that confer resistance to tetracyclines, sulfonamides and macrolides. The strains showed high diversity, where six were assigned to sequence type (ST)/2, and two were allocated to two new STs (ST/1411-1412). MLST analyses of 108 strains from Southeast Asia identified 19 sequence types (ST), and ST/2 was the most prevalent found in 62 strains. A broad range of AMR genes was identified mediating resistance to ß-lactams, including cephalosporins and carbapenems (e.g., *bla*OXA-51-like, *bla*OXA-23, *bla*ADC-25, *bla*ADC-73, *bla*TEM-1, *bla*NDM-1), aminoglycosides (e.g., *ant*(3”)-IIa, *aph*(3”)-Ib, *aph*(6)-Id, *arm*A and *aph*(3’)-Ia), phenicoles (e.g., *cat*B8), tetracyclines (e.g., *tet.*B and *tet*.39), sulfonamides (e.g., *sul*.1 and sul.2), macrolides and lincosamide (e.g., *mph*.E, *msr*.E and *aba*F). MLST and core genome MLST (cgMLST) showed an extreme diversity among the strains. Several strains isolated from different countries clustered together by cgMLST; however, different clusters shared the same ST. Developing an action plan on AMR, increasing awareness and prohibiting the selling of antibiotics without prescription must be mandatory for this region. Such efforts are critical for enforcing targeted policies on the rational use of carbapenem compounds and controlling AMR dissemination and emergence in general.

## 1. Introduction

*Acinetobacter* (*A*.) *baumannii* is a notorious Gram-negative pathogen associated with a multitude of severe nosocomial infections and high mortalities in intensive care units (ICUs) [1]. The pathogen is known to cause ventilator-associated pneumonia, bloodstream, skin and urinary tract infections and secondary meningitis [2,3]. *A. baumannii* is intrinsically resistant to many antibiotics and may acquire resistance via mutational changes in chromosomal structure and through horizontal gene transfer [4]. The spread and dissemination of multidrug-resistant (MDR) and extremely drug-resistant (XDR) *A. baumannii* have become a public health concern in both developing and developed countries. The prevalence of resistance towards last-resort antibiotics such as carbapenems and colistin is increasing globally [4,5]. The treatment of carbapenem-resistant *A. baumannii* (CRAb) is becoming a global challenge. CRAb is emerging worldwide, and the majority of these isolates often show MDR or XDR patterns [6,7]. They are also associated with an increased length of stay at hospital ICUs [8]. CRAb is associated with increased mortalities in patients with bloodstream infections in low- and middle-income countries [9]. It is considered one of the most common pathogens of nosocomial infections in Southeast Asia [10].

Vietnam is one of the Southeast Asian countries with the highest resistance prevalences amongst Gram-negative pathogens in the Asia-Pacific region [11]. It is considered the “hottest” spot of MDR *A. baumannii* in Asia. *A. baumannii* was considered one of the most frequent causes of ventilator-associated respiratory infection [12,13,14] and secondary meningitis [15] in Vietnamese ICUs. From 2008 to 2011, 101 clinical *A. baumannii* strains were isolated from ICU patients in two medical settings in Vietnam [16]. Between 2012 and 2014, 252 *Acinetobacter* spp. were isolated from patients admitted to three hospitals in southern Vietnam. Among them, 160 were confirmed as *A. baumannii* [17]. Between 2014 and 2015, *A. baumannii* posed serious therapeutic problems in ICU patients at a major tertiary hospital in Ho Chi Minh City [18]. Several separate studies have been carried out on *A. baumannii* in Southeast Asian countries and showed increases in the general distribution of *A. baumannii* in hospitals in Vietnam [12,17,18,19,20], Malaysia [21], Thailand [22], the Philippines [23] and Indonesia [24]. However, no comparison of the circulating strains and no multinational transboundary studies have been carried out to investigate the resistance profiles, MLST and the lineage of *A. baumannii* in the Southeast Asia region.

Thus, the current study aimed to analyze the whole-genome sequencing (WGS) data of carbapenem-resistant *A. baumannii* isolates obtained from Vietnamese patients and compare their AMR genes, MLST types and genetic diversity with published genomes of strains from Southeast Asia.

## 2. Results and Discussion

### 2.1. Whole-Genome Sequencing Data, MLST and cgMLST Analysis

Genome sequencing of 11 Vietnamese *A. baumannii* isolates yielded an average read length of 262,090 per isolate (range 1,137,862–2,537,360). The isolates’ mean coverage was 105.9-fold (range from 72-fold to 149-fold) (Table S1). To check for possible contamination and accurate species identification, the software Kraken2 was used, which classifies each read (or contig) [25]. At the genus level, the first match for all isolates was always “*Acinetobacter*”, on average, 97.61% of the reads (maximum 99.18%, minimum 89.61%). At the species level, the first match for all 11 isolates was always “*Acinetobacter baumannii*”, on average, 78.24% of the reads. Genome assembly yielded an average genome size of 4,070,357 bp with a minimum of 3,792,190 bp. The GC content was, on average, 38.97%. The mean N50 of the 11 assembled genomes was 88.347 bp (range 41,904–184,445 bp) (Table S2). MLST analyses of eleven Vietnamese *A. baumannii* strains based on the Pasteur scheme allocated nine strains into a distinct ST. Six strains were assigned to ST/2, two were assigned to ST/571 and one was assigned to ST/164. Two isolates were allocated into two new STs (ST/1411-1412) (Table 1).

MLST analysis confirmed the considerable genetic diversity of *A. baumannii* in the investigated strains from Southeast Asia. Six strains were removed as they did not fit our quality criteria. MLST analyses based on the Pasteur scheme identified 19 sequence types (ST) for 107 strains, and a strain from Thailand could not be assigned to a distinct sequence type due to new alleles. ST/2 was the most prevalent sequence type circulating in the Southeast Asian countries. It was found in 62 strains isolated from humans from Vietnam, Myanmar, Thailand, Singapore and Malaysia, followed by ST/164 and ST/16, which were found in nine and six strains, respectively. A strain obtained from the soil in Malaysia was assigned to ST/46.

The MLST analyses showed that more than half of the human strains (62 out of 107) in the current study were assigned to ST/2 (Pasteur). ST/2 belongs to the international clone II and is the most dominant type globally [26]. In previous studies, most of the CRAb isolates were found belong to the ST2 lineage in strains isolated from Thailand [22] and Myanmar [27] and among strains isolated from all five countries that contributed to this study. From 2008 to 2011, 101 clinical *A. baumannii* strains were isolated from patients in two medical settings in Vietnam. Most of the *A. baumannii* isolates obtained from a hospital in Hanoi were ST/91 and ST/231, whereas almost all strains from a hospital in Ho Chi Minh City were ST/136, ST/195 and ST/254 [16]. ST/1, ST/575 and ST/109 were found only in Myanmar, while ST/46, 220, 360, 374 and ST/739 were found only in Malaysia. ST/52 and ST/215 were found only in Thailand, while ST/129 was found only in Taiwan (Table 1). The published knowledge on the general distribution of STs of *A. baumannii* in the population of Southeast Asia is scarce. These findings are, moreover, in agreement with those of Gaiarsa et al., who demonstrated that the Pasteur scheme is more appropriate for epidemiological studies of *A. baumannii* [28] and proposed it to be the scheme of choice in parallel with cgMLST.

In the current study, we applied the cgMLST scheme included in the SeqSphere+ Ridom software tools, using a gene-by-gene approach to compare and describe the geographical relationship and the lineage among *A. baumannii* strains from Vietnam and Southeast Asia. The Vietnamese strains showed high diversity. Five strains appeared in three different clusters, and six strains were unique singletons. WGS showed a higher discriminatory power than conventional typing methods [6]. It allows a more precise trackback analysis of the strains, particularly when analyzing carbapenem-resistant *A. baumannii* isolates, which threaten the global healthcare system. We observed 14 different clusters (1 to 14) using cgMLST SeqSphere+ analysis. Clusters 1 and 5 contained isolates from Thailand and Myanmar. However, isolates were obtained from different countries and clustered in two distinctive clusters but shared the same Pasteur sequence type (ST/2). Cluster 11 contained one strain from Thailand and one from Vietnam and shared the same Pasteur sequence type (ST/164). Our analyses demonstrate that the strains are highly diverse. However, there was a good correlation between MLST and cgMLST clusters. All strains in the same cluster were assigned to the same ST. The predominant type ST/2 based on the Pasteur scheme could be sub-divided into eleven different clusters and distinct lineages when cgMLST was employed. These results show a superior discriminatory ability of cgMLST compared to the conventional MLST [29].

### 2.2. Antibiotic Susceptibility Testing (AST) and AMR Determinants of Vietnamese Strains

Antibiotic resistance is a serious problem in the Asia-Pacific region, and *A. baumannii* strains are among the most accounted for Gram-negative bacteria (GNB) causing urinary tract infections in this region [30]. Ten out of eleven *A. baumannii* strains originating from Vietnam were MDR, displayed resistance to fluoroquinolones (ciprofloxacin and levofloxacin), carbapenems (imipenem, meropenem and ertapenem), third-generation cephalosporins (cefotaxime, ceftazidime, ceftazidime/avibactam and ceftolozane/tazobactam), fourth-generation cephalosporins (cefepime), piperacillin, piperacillin/tazobactam, chloramphenicol and fosfomycin and were susceptible only to colistin. Resistance to the tetracycline derivative tigecycline (MIC = 1 µg/mL) and trimethoprim/sulfamethoxazole (MIC > 4/76 µg/mL) was seen in nine strains (Table S3). All MDR strains (*n* = 10) were carbapenem-resistant with MIC values of >8 µg/mL to imipenem (IMP) and >0.5 µg/mL to ertapenem (ERT). The MIC values to meropenem (MER) ranged from 32 to 128 µg/mL, higher than the MIC value (16 µg/mL) reported for the *A. baumannii* DMS06669 strain isolated in a Vietnam hospital [31]. From 2012 to 2014, resistance to cephalosporins, fluoroquinolones and carbapenems was >90% among 904 *A. baumannii* isolates from patients with hospital-acquired or ventilator-associated pneumonia in Vietnam [13], while colistin (MIC90, ≤0.25 mg/L) and tigecycline (MIC90, 4 mg/L) showed appreciable activity against *A. baumannii.* MIC90 for meropenem and imipenem was >32 mg/L in more than 80% of 74 strains isolated from tracheal aspirate specimens taken from patients with suspected ventilator-associated pneumonia from January 2011 to June 2012 [32]. All MIC values of tested antibiotics in the current study are shown in Table 2. One strain showed susceptibility to almost all tested antibiotics. It may has been isolated from a patient from rural areas where the use of antibiotics is seldom because they always depend on medicinal plants in treatment, in contrast to other patients.

The genome of all strains (100%) harbored *ant*(3”)-IIa, which confers resistance to aminoglycosides, while the *arm*A gene was found in eight (72.7%) isolates, and *aph*(6)-Id and *aph*(3”)-Ib were found in seven (63.6%) isolates. All isolates harbored at least one of the *bla*_OXA-51_ variants and *bla*_ADC-25,_ which confer resistance to ß-lactams, while *bla*_OXA-23_ and *bla*_TEM-1_ were found in nine (81.8%) and six (54.5%) isolates, respectively. More than half of the Vietnamese strains harbored genes that confer resistance to tetracyclines, sulfonamides and macrolides (Table S4).

The prevalence of MDR/CRAb was very high. The results are in agreement with previous reports: most CRAb strains display resistance to at least one compound in three or more antimicrobial categories and are designated as MDR [6,7]. Resistance against imipenem was seen in 91.6% of XDR *A. baumannii* strains isolated from patients in three hospitals in southern Vietnam between 2012 and 2014 [17]. A strain resistant to all tested classes of antibiotics except ciprofloxacin and colistin was isolated from the sputum of a patient with hospital-acquired pneumonia at the general hospital of Dong Nai [19]. Examination of 79 strains recovered from patients with pneumonia in Thong Nhat Dong Nai General Hospital showed carbapenem resistance and MDR in 80% and 90% of isolates, respectively [33]. This study highlights the very high prevalence of MDR/CRAb in the General Hospital of Phutho, Hanoi. Clinically, *A. baumannii* has become a notorious nosocomial pathogen worldwide [26], and the emergence of MDR/CRAb has serious consequences in the healthcare system in Southeast Asia [10]. The general distribution of *A. baumannii* is increasing in hospitals in Vietnam [19], Thailand [22,34], the Philippines [23], Malaysia [21], Indonesia [24] and Taiwan [35]. Our results support previous data from Southeast Asian hospitals, where a substantial increase in the MDR/CRAb isolation rate was demonstrated [10]. Identification of the genetic determinants associated with carbapenem resistance in *A. baumannii* is helping to explain the continuous selection and ongoing transmission within the healthcare system of Vietnam and other Southeast Asian countries.

### 2.3. Predicted Phenotype and AMR Determinants of A. baumannii from Southeast Asia

To compare the findings of AMR determinants in Vietnam to neighboring countries, we downloaded and analyzed *A. baumannii* sequence data from Southeast Asia. The frequency and percentage of resistance genes conferring specific antibiotic resistance were identified in 108 *A. baumannii* whole genomes. Our WGS approach identified different AMR genes, among which at least 47 genes confer resistance to ß-lactams, 18 to aminoglycosides, 8 to phenicoles, 4 to tetracyclines, 3 to sulfonamides and 3 to macrolides and lincosamide (Table S4).

#### 2.3.1. Resistance to β-Lactams

In total, forty-seven AMR genes mediating resistance to ß-lactams, including cephalosporins and carbapenems, were identified. The Ambler class D β-lactamases were present in almost all strains. The variants of the intrinsic *bla*_OXA-51-like_ carbapenemase gene were found in 103 (95.5%) strains; *bla*_OXA-66_ was the most frequent and was found in 68 (61%) isolates, followed by *bla*_OXA-91,_ *bla*_OXA-402_ and *bla*_OXA-64_. Five isolates (4.5%) were devoid of *bla*_OXA-51-like_. The *bla*_OXA-23_ variant was found in 90 (83%) isolates, and *bla*_OXA-58_ was found in 13 (12%) isolates. In terms of Ambler class A β-lactamases, *bla*_TEM-1_ was found in 55 (51%) isolates, followed by *bla*-_PER-_ in 8 (7.5%) isolates, *bla*-_CARB-16_ in 5 isolates, *bla*-_VEB-21_ in 4 isolates and *bla*-_SHV-5_ in 1 strain. Regarding Ambler class B β-lactamases, *bla*_NDM-1_ was found in nine (8%) isolates, and *bla*_IMP-14_ was found in two strains. Sixteen *Acinetobacter*-derived cephalosporinase *bla*_ADC_ variants of the Ambler class C β-lactamases were identified. The *bla*_ADC-25_ variant was the most frequent variant and was detected in all isolates (100%), followed by *bla*_ADC-73_ in 50 (46%) isolates_,_ *bla*_ADC-52_ in 8 isolates, *bla*_ADC-30_ and *bla*_ADC-26_ in 7 isolates and *bla*_ADC-76,_ *bla*_ADC-169_ and *bla*_ADC-199_ in 6 isolates (Table S4).

Four Ambler classes of β-lactamases (i.e., classes A, B, C and D) were identified in the current study. Various resistance genes conferring resistance to carbapenems and cephalosporins were found in *A. baumannii* isolated from Southeast Asia. The *bla*_OXA-23_ and *bla*_OXA-51-like_ variants were among the most frequent AMR genes identified. Both are currently spreading on plasmids and associated with resistance to all β-lactam compounds, including carbapenems [21,36]. The ADC beta-lactamases are cephalosporinases with extended-spectrum resistance to cephalosporins. All strains harbored *bla*_ADC-25_, and approximately half of the strains (46%) harbored *bla*_ADC-73_, which are considered significant determinants responsible for cephalosporins resistance in *A. baumannii* [37]. More than half of the strains (51%) harbored *bla*_TEM-1_. It encodes a class A β-lactamase and has been found in CRAb strains from different regions worldwide [38,39,40].

#### 2.3.2. Resistance to Aminoglycosides

In the 108 analyzed strains, 18 AMR genes conferring resistance to aminoglycosides were identified. Aminoglycoside-modifying enzymes (AMEs), including acetyltransferases (*AAC*s), methyltransferase (*arm*A), phosphotransferases (*APH*s) and nucleotidyltransferases (*ANT*s), were identified. The new subclass of intrinsic aminoglycoside nucleotidyltransferase, *ANT*(3”)-IIa, was widely distributed and was found in almost all strains (99%), followed by the intrinsic aminoglycoside O-phosphotransferase *aph*(6)-Id and *aph*(3”)-Ib found in 83 (77%) strains. To date, over a hundred AMEs have been described, and AACs represent the largest group of AMEs [41]. In the current study, AAC genes were detected in 12% of the isolates, while *ANT* and *APH* genes were distributed in almost all isolates (Table 3). Moreover, the intrinsic aminoglycoside methyltransferase (MET) *arm*A was found in 73 (76.5%) isolates. *Arm*A is the most important class of plasmid-mediated MET enzymes that confer resistance to gentamicin in *Enterobacteriaceae* [42]. Aminoglycosides are broad-spectrum antibiotics used against a wide range of infections caused by Gram-negative bacteria in clinical settings. However, their efficacy has been reduced by resistance development [7,43]. This finding highlights the diversity of aminoglycoside-resistant *A. baumannii* strains mediated by *ANT*s, *APH*s, *arm*A and AACs of AMEs in Southeast Asian countries.

#### 2.3.3. Resistance to Phenicoles, Tetracyclines, Macrolides, Sulfonamides and Rifamycin

At least eight genes encoding resistance to phenicoles (*cml*A, *cat*B8, *cml*A1, *flo*R, *cml*A5, *cat*B3, *cat*A1 and *cml*A6) were identified. *Cat*B8 was the most frequent and found in 14 (13%) isolates, followed by *cml*A in 6 isolates and *flo*R in 4 isolates. Most *A. baumannii* isolates are intrinsically resistant to chloramphenicol [44]. However, genes encoding resistance to phenicoles were found only in 13% of the isolates. Four genes encoding resistance to tetracycline (*tet*.B, *tet*.39, *tet*.A, *tet*.M) were identified. *Tet*.B was identified in 79 (73%) isolates, and *tet*.39 was identified in 15 (14%) isolates. *Tet.*B is a tetracycline efflux protein that confers resistance to tetracycline but not to tigecycline [45]. In the current survey, it was found in the majority of strains. Two genes encoding macrolide resistance were identified. *Mph*.E and *msr*.E were identified in 77% and 79% of the isolates, respectively. Three genes encoding resistance to sulfonamides (*sul*1, *sul*2 and *sul*3) were identified. *Sul*2 and *sul*1 variants were found in 66% and 29% of the strains, respectively, while *sul*3 was found in one isolate. Both *sul*2 and *sul*1 are mediated by transposons and plasmids [46]. The presence of one or both genes in *A. baumannii* isolates might confer resistance to trimethoprim/sulfamethoxazole. The *arr*-2 gene confers resistance to rifampicin was found in 14 (13%) strains (Table 3). Several resistance mechanisms for different antibiotic classes exist in *A. baumannii* [47] and were found in the current study. Genes encoding resistance to macrolides, tetracyclines, sulfonamides and phenicoles were seen in 77%, 73%, 66% and 13% of the isolates, respectively. The circulation of genes at such high frequency in Southeast Asia is alarming and highlights the urgent need to take effective control measures.

#### 2.3.4. Antibiotic Efflux Pumps

Four categories of efflux pumps were found in *A. baumannii *isolates of Southeast Asian origin, including the resistance-nodulation-division (RND) superfamily, the major facilitator superfamily (MFS), the multidrug and toxic compound extrusion (MATE) family and the small multidrug resistance (SMR) family transporters. Among these different pumps, the MFS transporter (*amv*A), RND (*ade*FGH, *ade*IJK and *ade*L), SMR (*abe*S) and MATE (*abe*M) were most frequent and found in almost all isolates (99–100%). RND efflux pump-coding genes (*ade*N, *ade*R, *ade*S and *ade*AB) were found in 92.2%–94.5% of the strains (Table S4). Resistance mediated by antibiotic efflux pump-encoding genes is well documented in *A. baumannii* [7,20]. Efflux pumps play significant roles in developing AMR in *A. baumannii* [48]. The chromosomally encoded tripartite efflux pump *ade*ABC is a worldwide-distributed RND superfamily efflux pump in *A. baumannii* and was found in approximately 80% of the clinical strains. The overexpression of *ade*ABC efflux pumps is mainly associated with reduced susceptibility to tigecycline [49], fluoroquinolones, tetracycline, chloramphenicol and erythromycin, confers resistance to aminoglycosides [50,51] and may contribute resistance to carbapenems [39]. The circulation of numerous efflux pumps with high frequency suggests a significant rise in *A. baumannii* antibiotic resistance in Southeast Asia. *Acinetobacter baumannii Aba*F was found in 104 (96%) strains isolated from all contributing countries. *Aba*F is a major facilitator superfamily (MFS) antibiotic efflux pump interfering with protein synthesis. Its expression in *E. coli* increases resistance to fosfomycin. The high frequency of resistance to fosfomycin was seen in the tested strains and in a previous study on *A. baumannii* [7]. It has been reported that the *aba*F gene is involved in fosfomycin resistance in *A. baumannii* and plays a role in biofilm formation and virulence mechanisms [52].

### 2.4. Acquired Resistance in A. baumannii of Southeast Asian Origin

Examination of all strains in the current study, either sequenced strains from Vietnam or downloaded genomes from PlasmidFinder [53] and Platon [54] as two different tools to investigate the potential presence of plasmids, failed to detect plasmids or plasmid replicons in all strains, except for one strain from Myanmar which harbored only two replicons (Col.MG828._1 and Col8282_1). This information is included in Table S5. Moreover, the comprehensive ResFinder server [55] was used to investigate the potential acquired AMR genes in the *A. baumannii* strains. ResFinder can identify acquired genes and chromosomal mutations mediating AMR in a total or partial DNA sequence. Several genes encoding resistance to aminoglycosides, β-lactams, tetracycline, sulfonamides and macrolides were found. The *Acinetobacter*-derived cephalosporinase *bla*_ADC.25_ conferring resistance to cephalosporin was identified in all isolates (100%), and the two genes conferring resistance to carbapenems, *bla*_OXA-23_ and *bla*_OXA-51-like_ (*bla*_OXA-66_ variant), were detected in 83% and 61% of the strains, respectively. Moreover, different variants of *bla*_TEM_, *bla*_CARB_, *bla*_IMP_, *bla*_VEB_, *bla*_PER_, *aph*.6.Id, *aph*.3.Ia, *aph*.3.Ib, *tet*, *sul* and *arm*A were identified using the ResFinder database. The presence of various plasmids in the genome of *A. baumannii* [56] and its ability to acquire foreign DNA [57,58] enhance the acquisition of AMR genes. Several reports suggested that mobile genetic elements play significant roles in the horizontal transfer of AMR genes in *A. baumannii*, particularly genes that confer resistance to aminoglycosides, chloramphenicol and tetracycline [59,60,61]. Identification of a wide variety of AMR genes by ResFinder in the current study highlights the role of horizontal gene transfer in the development of resistance in *A. baumannii* in Southeast Asia.

## 3. Materials and Methods

### 3.1. Identification of Bacterial Isolates and Antibiotics Susceptibility Testing (AST)

In total, eleven non-repetitive *A. baumannii* strains isolated from Vietnamese patients at the General Hospital of Phutho, Hanoi, were received by the Institute of Bacterial Infections and Zoonoses (IBIZ, Jena) for species confirmation and typing. The strains were isolated from blood, sputum, CSF and abscess samples of patients admitted to the hospital in 2017. The agreement for receiving the genetic samples according to the Nagoya Protocol was obtained from the Natural Resources and Environment Minister. No additional ethical approval was required. All strains were identified at species level using a combination of matrix-assisted laser desorption/ionization mass spectrometry (MALDI-TOF MS) with a log value of >2.300 and the intrinsic *bla*_OXA-51-like_-PCR [62]. The minimum inhibitory concentration (MIC) was determined by the broth microdilution method using an automated MICRONAUT-S system (Micronaut, MERLIN Diagnostics GmbH, Bornheim-Hersel Germany) according to the manufacturer’s instructions. The results were evaluated as susceptible, intermediate and resistant automatically with the built-in MICRONAUTS software. The MIC values for a panel of the 18 antibiotics were interpreted according to the Clinical and Laboratory Standards Institute (CLSI) breakpoint guidelines available for *A. baumannii* as previously described [7].

### 3.2. Whole-Genome Sequencing and Collection of Sequence Data from Southeast Asia

DNA extraction was performed using the High-Pure template preparation kit (Roche Applied Sciences, Mannheim, Germany) according to the manufacturer’s instructions. The library preparation and paired-end sequencing on an Illumina MiSeq sequencer were performed for the 11 Vietnamese *A. baumannii* strains as previously described [7]. Briefly, the Nextera XT DNA Library Prep Kit (Illumina, Inc., San Diego, CA, USA) was utilized to prepare the sequencing library, followed by paired-end sequencing on an Illumina MiSeq sequencer (Illumina, San Diego, CA, USA).

Raw sequencing data were downloaded from NCBIs’ Sequence Read Archive (https://www.ncbi.nlm.nih.gov/sra/?term, accessed on 1 November 2020) according to the following criteria: Search for species [*Acinetobacter baumannii*] with the geo_loc_name_country_continent [Asia] provided 1025 genomes. The results were filtered, and the search items following the Library Layout [paired], Library Source [genomic] and Platform [Illumina] were eligible for inclusion. Raw sequence data of all *A. baumannii* strains belonging to the search criteria from Southeast Asian countries were included in the study. Data of 103 *A. baumannii* strains were extracted. Of these, 49 *A. baumannii* genomes were from Thailand (BioProjects: PRJNA623108, PRJNA627433, PRJNA647677 and PRJNA389557], 38 were from Myanmar (BioProject: PRJDB8528), 11 were from Malaysian BioProjects (PRJNA565663 and PRJNA185400), four were from a Singapori BioProject (PRJNA627433), and one was from a Taiwanese BioProject (PRJNA627433). No genomes were found for Vietnam, Indonesia, the Philippines, Cambodia, Laos, Brunei and Timor-Leste (Table 4).

### 3.3. Bioinformatic Data Analysis

Data analysis of sequences from eleven samples sequenced within this study and downloaded sequences was performed with the pipeline WGSBAC (v2.0.0) [7,63,64]. In short, raw sequencing data quality was controlled by WGSBAC with FastQC (v. 0.11.5) [65], and coverage was determined. Shovill (v. 1.0.4), based on SPAdes (v3.14.0) [25], was used for assembly. Assembly quality was checked with QUAST (v. 5.0.2) [66]. Kraken 2 (v. 2.0.7 beta) [67], in combination with the database MiniKraken (v2), was used to classify reads and assemblies and to check for contamination. In silico determination of classical multilocus sequence typing (MLST) was performed based on the assembled genomes using mlst software (v. 2.16.1) according to the *Acinetobacter baumannii*#2 scheme published by Diancourt and coworkers and referred to as the Pasteur scheme [68]. For antimicrobial resistance profiling and determination of AMR genes, the databases from NCBI AMR Finder Plus [69], ResFinder [55] and CARD [70] were used. Core-Genome MLST (cgMLST) was performed by employing the software Ridom SeqSphere+, version 5.1.0 (Ridom GmbH, Münster, Germany) [71]. PlasmidFinder [53] and Platon [54] were used to investigate the potential presence of plasmids and plasmid replicons.

## 4. Conclusions and the Way Forward

Southeast Asia encompasses eleven countries with a wide diversity in history, culture and religion: Brunei, Myanmar (Burma), Cambodia, Timor-Leste, Indonesia, Laos, Malaysia, the Philippines, Singapore, Thailand and Vietnam. According to the Southeast Asia Infectious Disease Clinical Research Network, a previous multinational, multicenter cross-sectional study showed that *A. baumannii* was among the causative pathogens of sepsis in South Asian countries [72] and that CRAb was the most common nosocomial pathogen associated with infection in ICUs in this region [10]. In silico analysis of 108 whole genomes of *A. baumannii* strains isolated from Southeast Asia successfully assigned 107 strains into distinct STs using the Pasteur scheme. MLST analysis confirmed the considerable diversity of *A. baumannii,* and ST/2 was the most prevalent sequence type. The strains harbored a wide variety of AMR genes mediating resistance mostly to β-lactams (including cephalosporins and carbapenems), aminoglycosides, phenicoles, tetracyclines, sulfonamides and macrolides. However, the strain resistance phenotype is unknown except for Vietnamese strains. Several antibiotic resistance mechanisms for various antibiotic classes were observed, including β-lactamases, aminoglycoside-modifying enzymes, permeability defects, alteration and replacement of antibiotic target sites, enzymatic inactivation, multidrug efflux pumps and acquisition of AMR genes. *A. baumannii* combines clonal spread with high genetic flexibility. The clonal spread of ST/2 is obvious, but there is a lot of evolutionary dynamic outside ST/2 and, most concerning, also within ST2. Aminoglycosides mediating resistance genes *ant*(3”)-IIa, *aph*(6)-Id, *arm*A and *aph*(3”)-Ib, and *bla*_OXA-64_, *bla*_OXA-23_, *bla*_TEM-1_ and *bla*_ADC-73_ genes mediating resistance to β-lactams, as well as *tet*.B mediating resistance to tetracyclines, are the highest variable genes within ST/2. These might be genes playing a role in improving the adaptation to the environment. Therefore, both decreased antibiotic consumption and infection prevention and control (IPC) (mainly tackling the clinical component of the spread) are required to counteract this pathogen’s spread.

In general, the prevalence of MDR increases due to limited infection control measures and the lack of antimicrobial stewardship teams in healthcare settings. Moreover, antibiotics are sold without a prescription in rural and urban pharmacies in many developing countries, including Southeast Asian countries [73]. Notably, awareness of antibiotic resistance is missing, particularly in rural areas [73]. Uncontrolled travel throughout this region and transboundary trade of animals and foodstuffs have contributed to increasing AMR prevalence. Resistance to carbapenems is increasingly being reported in Southeast Asia health facilities where the antibiotic is not routinely used and is emerging despite its restricted uses due to high costs. A way forward for this region is to design multinational collaborative efforts geared towards investigating the molecular epidemiology of CRAb and its burden on healthcare systems and understanding the underlying genetic mechanisms associated with resistance to carbapenems. In 2013, the Vietnamese Ministry of Health was the first ministry in the WHO Western Pacific region to develop a national action plan on AMR. The antimicrobial resistance reference laboratory and surveillance program were initiated in Vietnam in 2017 [74]. However, within a region characterized by open borders, an environment with high prevalences of AMR organisms and patient self-treatment, a multinational transboundary surveillance program is urgently needed [75]. Multinational unified antimicrobial stewardship and broad-scale collaboration would ultimately enable the persons in charge to identify existing hotspots, possible reservoirs and possible practices, cultures and attitudes that may predispose different communities to CRAb infections. Control of selling antibiotics without prescription and increasing awareness are required. Implementing the Vietnamese way, i.e., developing a national action plan on AMR and establishing an antimicrobial resistance reference laboratory, is a supreme priority. Such efforts would be critical in creating targeted policies on carbapenem compounds’ rational use and controlling AMR’s dissemination and emergence in general. This will be significant because carbapenems are expected to become cheaper and readily available in the future in most countries.

## Figures and Tables

**Table 1:** Strain types (ST) of 108 *A. baumannii* strains isolated in Southeast Asia according to the Pasteur scheme.

MLST	Frequency	Country
ST/2	62	Vietnam (6), Myanmar (19), Thailand (33), Singapore (3), Malaysia (1)
ST/164	9	Vietnam (1), Myanmar (5), Thailand (3)
ST/16	6	Myanmar (2), Thailand (4)
ST/23	4	Myanmar (3), Thailand (1)
ST/25	4	Myanmar (3), Thailand (1)
ST/1	3	Myanmar (3)
ST/215	3	Thailand (3)
ST/571	3	Vietnam (2), Thailand (1)
ST/374	2	Malaysia (2)
ST/575	2	Myanmar (2)
ST/46	1	Malaysia, soil (1)
ST/52	1	Thailand (1)
ST/109	1	Myanmar (1)
ST/129	1	Taiwan (1) *
ST/220	1	Malaysia (1)
ST/360	1	Malaysia (1)
ST/739	1	Malaysia (1)
ST/1411	1	Vietnam (1), new ST
ST/1412	1	Vietnam (1), new ST
ND	1	Thailand (1)
20	108	Total

* Taiwan is part of the area of Greater China and is used as an example of a trading country with Southeast Asian countries.

**Table 2:** The MIC (µg/mL) for the 11 sequenced Vietnamese *A. baumannii* strains as evaluated with MICRONAUT software.

ID	CIP	LEV	AMK	COL	CMP	FOS	TGC	T/S	PIP	PIT	CTX	CAZ	CAA	CTA	CEP	IMP	MER	ERT
18Y0059	>2	>2	>32	≤1	>16	>64	=1	>4/76	>16	>64/4	>2	>128	>16/4	>8/4	=128	>8	=64	>0.5
18Y0060	>2	>2	>32	≤1	>16	>64	=1	>4/76	>16	>64/4	>2	>128	>16/4	>8/4	=128	>8	=64	>0.5
18Y0061	>2	>2	>32	≤1	>16	>64	=1	4/76	>16	>64/4	>2	>128	>16/4	>8/4	=64	>8	=64	>0.5
18Y0064	>2	>2	>32	≤1	>16	>64	=1	>4/76	>16	>64/4	>2	>128	>16/4	>8/4	=64	>8	=128	>0.5
18Y0065	>2	>2	>32	≤1	>16	>64	=1	=2/38	>16	>64/4	>2	>128	>16/4	>8/4	=128	>8	=64	>0.5
18Y0066	>2	>2	>32	≤1	>16	>64	0.5	>4/76	>16	>64/4	>2	=64	=16/4	>8/4	=64	>8	=128	>0.5
18Y0067	>2	>2	>32	≤1	>16	>64	=1	>4/76	>16	>64/4	>2	=64	=16/4	>8/4	=64	>8	=64	>0.5
18Y0068	≤0.25	≥0.5	≤4	≤1	>16	>64	≤0.25	=4/76	≤8	≤4/4	=2	≤1	≤1/4	≤1/4	≤1	≤1	≤0.125	=0.25
18Y0072	>2	>2	≤4	≤1	>16	>64	≤0.25	≤1/19	>16	>64/4	>2	>128	=16/4	>8/4	=128	>8	=32	>0.5
18Y0074	>2	>2	>32	≤1	>16	>64	0.5	>4/76	>16	>64/4	>2	>128	=16/4	>8/4	=32	>8	=64	>0.5
18Y0075	>2	>2	>32	≤1	>16	>64	=1	=4/76	>16	>64/4	>2	>128	>16/4	>8/4	=128	>8	=64	>0.5

The minimum inhibitory concentration (MIC), ciprofloxacin (CIP), levofloxacin (LEV), amikacin (AMK), colistin (COL), chloramphenicol (CMP), fosfomycin (FOS), tigecycline (TGC), trimethoprim/sulfamethoxazole (T/S), piperacillin (PIP), piperacillin/tazobactam (PIT), cefotaxime (CTX), ceftazidime (CAZ), ceftazidime/avibactam (CAA), ceftolozane/tazobactam (CTA), cefepime (CEP), imipenem (IMP), meropenem (MER) and ertapenem (ERT).

**Table 3:** AMR genes detected in 108 whole-genome sequences of *A. baumannii* originating from Southeast Asia.

Antibiotic Class	AMR Resistance Genes		Mechanism	Predicted Phenotype	Origin of Strains
	Gene Family	Frequency (%)			
Aminoglycosides	*ant(3“)-IIa*	107 (99%)	NUT: Nucleotidyltransferase	Streptomycin, spectinomycin	Viet, Myan, Thai, Sing, Mala, Tiaw
	*aph(3“)-Ib*	83 (77%)	PHT: Phosphotransferase	Streptomycin	Viet, Myan, Thai, Sing, Mala, Tiaw
	*aph(6)-Id*	83 (77%)	PHT: Phosphotransferase	Streptomycin	Viet, Myan, Thai, Sing, Mala, Tiaw
	*armA_1*	73 (63.5%)	MET: Methyltransferase	Gentamicin	Viet, Myan, Thai, Sing, Mala, Tiaw
	*aph(3’)-Ia*	53 (49%)	PHT: Phosphotransferase	Kanamycin	Viet, Myan, Thai, Sing, Mala, Tiaw
	*aadA1*	22 (20%)	NUT: Nucleotidyltransferase	Streptomycin	Viet, Myan, Thai, Sing, Tiaw
	*aph(3’)-Via*	15 (14%)	PHT: Phosphotransferase	Amikacin, kanamycin	Viet, Myan, Thai,
	*ant(2“)-Ia*	14 (13%)	NUT: Nucleotidyltransferase	Gentamicin, kanamycin	Viet, Myan, Thai
	*aac(6’)-Ib*	12 (11%)	ACT: Acetyltransferase	Gentamicin	Viet, Myan, Thai, Tiaw
	*aac(3)-IId*	11 (10%)	ACT: Acetyltransferase	Gentamicin	Myan, Thai
β-lactams	*bla*OXA-51-like	103 (95.5%)	Ambler class D β-lactamases	β-lactam (carbapenem)	Viet, Myan, Thai, Sing, Mala, Tiaw
	*bla*OXA-66	68 (61%)	blaOXA-51 variant	β-lactam (carbapenem)	Viet, Myan, Thai, Sing, Mala, Tiaw
	*bla*OXA-91	10 (9%)	blaOXA-51 variant	β-lactam (carbapenem)	Viet, Myan, Thai
	*bla*OXA-23	90 (83%)	Ambler class D β-lactamases	β-lactam (carbapenem)	Viet, Myan, Thai, Sing, Mala, Tiaw
	*bla*OXA-58	13 (12%)	Ambler class D β-lactamases	β-lactam (carbapenem)	Viet, Myan, Thai, Mala
	*bla*TEM-1	55 (51%)	Ambler class A β-lactamases	β-lactam	Viet, Myan, Thai, Sing, Mala
	*bla*ADC-25	108 (100%)	Ambler class C β-lactamases	β-lactam (cephalosporin)	Viet, Myan, Thai, Sing, Mala, Tiaw
	*bla*ADC-73	50 (46%)	Ambler class C β-lactamases	β-lactam (cephalosporin)	Viet, Myan, Thai
	*bla*NDM-1	9 (8.5%)	Ambler class B β-lactamases	β-lactam (carbapenem)	Viet, Myan, Thai
Phenicoles	*cat*B8	14 (13%)	Enzymes inactivation	Chloramphenicol	Viet, Myan, Thai, Tiaw
Macrolide	*mph*.E.	83 (77%)	Enzymes inactivation	Macrolide	Viet, Myan, Thai, Sing, Mala, Tiaw
	*msr*.E.	85 (79%)	Antibiotic efflux	Macrolide	Viet, Myan, Thai, Sing, Mala, Tiaw
Sulfonamides	*sul*1	31 (29%)	Antibiotic target replacement	Sulfonamide	Viet, Myan, Thai, Sing, Mala, Tiaw
	*sul*2	71 (66%)	Antibiotic target replacement	Sulfonamide	Viet, Myan, Thai, Sing, Mala, Tiaw
Tetracyclines	*tet*.39.	15 (14%)	Antibiotic efflux	Tetracycline	Viet, Myan, Thai
	*tet*.B.	79 (73%)	Antibiotic efflux	Tetracycline	Viet, Myan, Thai, Sing, Mala, Tiaw
Rifamycin	*arr*-2	14 (13%)		Rifamycin	Myan, Thai,

Viet (Vietnam), Myan (Myanmar), Thai (Thailand), Sing (Singapore), Mala (Malaysia), Tiaw (Taiwan).

**Table 4:** Numbers, geographical origin and source of *A. baumannii* genomes investigated in the current study from Southeast Asia.

No.	Country	Geographical Location	No. of Strains	No. of Strains Analyzed	Source of Sequence
1	Thailand	Southeast Asia	49	49	NCBI
2	Myanmar	Southeast Asia	38	37	NCBI
3	Malaysia	Southeast Asia	11	7	NCBI
4	Vietnam	Southeast Asia	11	11	IBIZ/FLI
5	Singapore	Southeast Asia	4	3	NCBI
6	Indonesia	Southeast Asia	0	0	-
7	Philippines	Southeast Asia	0	0	-
8	Cambodia	Southeast Asia	0	0	-
9	Laos	Southeast Asia	0	0	-
10	Brunei	Southeast Asia	0	0	-
11	Timor-Leste	Southeast Asia	0	0	-
12	Taiwan	East Asia/Trade country	1	1	NCBI
Total			114	108	

## Data Availability

All study data are included in the article and supporting information. The data have also been submitted to the European Nucleotide Archive (ENA). The project accession number is PRJEB43552.

## Supplementary Materials

The following are available online at https://www.mdpi.com/article/10.3390/antibiotics10050563/s1. Table S1: The average read length and the coverage of 108 *A. baumannii* isolates from Southeast Asia. Table S2: The data of genome assembly of 11 *A. baumannii* isolates from Vietnam. Table S3: The results of antibiotic susceptibility testing of Vietnamese *A. baumannii* isolates. Table S4: Metadata, MLST, AMR genes and the predicted antibiotic classes of 108 *A. baumannii* isolates from Southeast Asia. Table S5: The results of PlasmidFinder for all *A. baumannii* strains provide some information about the presence of replicons.
